# Impact of Volume Control Biosensors on Blood Pressure During Haemodialysis: A Quasi-Experimental Study

**DOI:** 10.3390/healthcare13162060

**Published:** 2025-08-20

**Authors:** Veronica Gimeno-Hernan, Carla Perez-Ingidua, Ana Belen Rivas-Paterna, Natividad Codesal-Sanabria, Guillermo Perez-Duque, Ana Ascaso-del-Rio, Ismael Ortuño-Soriano

**Affiliations:** 1 Nursing Department, Faculty of Nursing Physiotherapy and Podology, Universidad CompluDtense de Madrid, 28040 Madrid, Spain; carlap05@ucm.es (C.P.-I.); ab.rivas@enf.ucm.es (A.B.R.-P.); iortunos@ucm.es (I.O.-S.); 2 Clinical Pharmacology Department, Instituto de Investigación Sanitaria del Hospital Clínico San Carlos (IdISSC), 28040 Madrid, Spain; a.ascasodelrio@gmail.com; 3 Clinical Research and Trials Unit, Instituto de Investigación Sanitaria del Hospital Clínico San Carlos (IdISSC), 28040 Madrid, Spain; 4 Nephrology Department, Hospital Universitario del Henares, 28040 Madrid, Spain; nati.codesal@gmail.com; 5 Nephrology Department, Hospital Clínico San Carlos, 28040 Madrid, Spain; guiperdu@gmail.com; 6 Pharmacology and Toxicology Department, School of Medicine, Universidad Complutense de Madrid, 28040 Madrid, Spain; 7 Health Care Research Group, Instituto de Investigación Sanitaria Hospital Clínico San Carlos (Iddisc), 28040 Madrid, Spain

**Keywords:** biosensor, hypotension, hemodialysis, vascular refilling, nurse

## Abstract

**Background:** Intradialytic hypotension is a common complication in haemodialysis, affecting up to 30% of sessions. It results from an imbalance between ultrafiltration and compensatory mechanisms, such as vascular tone and plasma refilling. Volume-controlled biosensors allow for the continuous monitoring of the haemoconcentration, enabling early detection and prevention of hypotension. **Methods:** A quasi-experimental study was conducted to assess the effectiveness of biosensors in reducing hypotensive episodes. Two biosensors were compared: the Blood Volume Monitor and the Haemomaster system. Data were collected over two four-month periods: before and after biosensor implementation. Nursing staff received specific training, and a protocol for consistent data collection was established. Informed consent was obtained from all eligible participants. The incidence of intradialytic hypotension was compared between sessions with and without biosensor use. Additionally, outcomes were analysed according to biosensor type. **Results:** A total of 2262 dialysis sessions from 22 patients were analysed. The cohort was 54.5% male, with a mean age of 60 years (SE = 21); 27.3% had diabetes and 81.8% had hypertension. Post-dilution haemodiafiltration was performed in 62.8% of sessions. Intradialysis hypotension occurred in 11.2% of sessions using biosensors compared to 14.0% without (*p* = 0.021). No significant difference was found between biosensor types (10.8% vs. 11.8%; *p* = 0.531), although device 1 reached a significantly lower critical blood volume (mean: 10 L; SE = 4 vs. 16 L; SE = 5; *p* = 0.000). **Conclusions:** Biosensor use was associated with fewer hypotensive episodes and greater haemodynamic stability. These findings support their integration into routine dialysis practice to improve treatment, safety, and individualised care.

## 1. Introduction

Intradialytic hypotension (IDH) is a common complication during haemodialysis sessions, affecting approximately 16–30% of treatments [1]. Recurrent IDH episodes have been associated with increased morbidity and mortality [2]. This condition primarily results from a reduction in circulating blood volume [3], most often caused by ultrafiltration rates that exceed the patient’s compensatory mechanisms, including vascular tone regulation and plasma refilling capacity. Both treatment-related factors (e.g., excessive ultrafiltration) and patient-specific conditions (e.g., autonomic dysfunction, and systolic or diastolic heart failure) contribute to this haemodynamic imbalance [4].

IDH is typically defined as a drop in systolic blood pressure greater than 20 mmHg, or a decrease of more than 10 mmHg in mean arterial pressure. This hypotensive response reflects insufficient plasma refilling during fluid removal, resulting in vascular underfilling despite ongoing interstitial fluid mobilisation [2].

Given the central role of hypovolaemia in IDH pathophysiology, automated biofeedback systems have been developed to monitor and regulate intravascular volume changes in real time, aiming to prevent critical drops in blood pressure [5]. These systems rely on biosensor technologies capable of adjusting dialysis parameters in response to the patient’s physiological and physical status [6,7]. The sensors detect changes in haematocrit or blood viscosity using dual-beam infrared spectroscopy or ultrasound velocity measurements [8]. A rapid decline in relative blood volume over a short interval is interpreted as a sign of insufficient vascular refill and excessive ultrafiltration, prompting compensatory adjustments in sodium conductivity or the ultrafiltration rate [9,10].

These biosensors enable continuous monitoring of the haemoconcentration throughout the dialysis session, facilitating early identification of patients at high risk for IDH and allowing for timely clinical intervention. Certain patient populations—such as individuals with diabetes mellitus (especially those with autonomic neuropathy), cardiac dysfunction (e.g., left ventricular hypertrophy, diastolic dysfunction, or heart failure), or significant interdialytic weight gain—are particularly vulnerable and warrant close surveillance [10].

Various strategies have been proposed to prevent or mitigate IDH by addressing its underlying pathophysiological mechanisms [11,12]. These include technical interventions (e.g., adjusting ultrafiltration rates, or modifying dialysate composition or temperature), pharmacological therapies aimed at enhancing sympathetic tone, and behavioural strategies such as withholding antihypertensive medication on dialysis days or avoiding intradialytic food intake [11,12,13]. In all cases, it remains essential to rule out non-dialysis-related causes of hypotension, such as pericardial effusion, infection, or myocardial ischaemia [11,12].

The most promising advances involve integrating real-time technologies that can modulate temperature, sodium conductivity, and ultrafiltration dynamically, thereby enhancing cardiovascular stability and maintaining vascular refill throughout the session [14].

Despite technological improvements, IDH remains a persistent challenge, largely due to the rapid fluid shifts intrinsic to intermittent dialysis therapy and the difficulty many patients face in adhering to sodium and fluid restrictions.

To evaluate the clinical effectiveness of biosensors, the objective of this study was to compare the incidence of hypotensive episodes in haemodialysis patients undergoing blood volume monitoring with biosensors versus standard clinical practice managed by the nursing staff.

## 2. Materials and Methods

A quasi-experimental study was conducted to evaluate the effectiveness of a biosensor-based system for controlling IDH in patients undergoing chronic haemodialysis. This design allowed for the comparison of two periods: one employing standard clinical practice and the other integrating biosensor technology into routine care.

The study was carried out in the haemodialysis unit of a tertiary hospital in Spain, specifically within the unit designated for chronic kidney disease patients.

The study population included all patients enrolled in the hospital-based chronic haemodialysis programme. Inclusion criteria were as follows: a diagnosis of stage V CKD, ongoing haemodialysis treatment in the hospital, interdialytic weight gain exceeding 1 L, age ≥ 18 years, and provision of written informed consent. Exclusion criteria comprised the following: interdialytic weight gain < 1 L, residual diuresis > 1.5 L in 24 h, and discontinuation of haemodialysis due to kidney transplantation, death, or a change in treatment modality.

A non-probabilistic convenience sampling approach was used, given the limited patient volume. Each dialysis session was considered as an individual sample unit. However, only 22 patients were ultimately included due to the availability of eligible participants during the study period. Although a total of 2262 dialysis sessions were analysed, these represent repeated measures within the same patients, which may affect the independence of observations and should be considered when interpreting the results.

The following two study periods were defined:

Period 0 (March–June 2022): Retrospective data from sessions conducted under standard clinical practice, which included conventional strategies for IDH management, such as adjustments to ultrafiltration volume, dialysate conductivity and temperature, and recommendations regarding food intake and antihypertensive medication.

Period 1 (December 2022–December 2023): Sessions incorporating biosensor-guided monitoring of relative blood volume changes, with the aim of anticipating and mitigating hypotensive episodes.

The primary outcome variable was the number of intradialytic hypotension events per session, defined as a ≥20 mmHg drop in systolic blood pressure or ≥10 mmHg decreases in mean arterial pressure. Secondary variables included the type of biosensor used, percentage change in relative blood volume, dialysis modality, prescribed ultrafiltration volume, session duration, age, sex, and comorbidities (diabetes mellitus, hypertension, and peripheral vascular disease), as well as antihypertensive medication use.

Data collection occurred in three phases. In Phase 1, all variables were recorded in an Excel database. Eight nurses with ≥5 years of clinical experience participated in data collection after receiving specific training. In Phase 2, additional theoretical and practical training was provided to nursing staff on haemodialysis monitors and biosensor operation. The devices employed included the FRESENIUS 5008 (Fresenius Medical Care, Waltham, MA, USA), and NIKISSO DBB-EXA (NIKKISO Co., Ltd., Tokyo, Japan) and DBB-07 monitors (Nikkiso Medical, Tokyo, Japan), all integrated with biosensor systems. In Phase 3, eligible patients were informed about the study, and written informed consent was obtained. Data were recorded during each session and validated monthly via clinical records. All sessions were supervised by medical staff and conducted using double-lumen catheters, arteriovenous fistulas, or vascular grafts.

Statistical analysis was performed using IBM SPSS Statistics v26. Qualitative variables were summarised using frequency distributions, while quantitative variables were described using measures of central tendency and dispersion. Group comparisons were conducted using Student’s *t*-test or ANOVA, and non-parametric comparisons were performed using the Mann–Whitney U test when appropriate. Categorical variables were compared using the chi-square test or Fisher’s exact test. A *p*-value < 0.05 was considered statistically significant.

As each patient contributed multiple dialysis sessions, the data included repeated measures, meaning that observations were not independent. However, repeated-measures models or hierarchical/mixed-effects modelling were not applied, which may have affected variance estimates and p-value precision.

### Ethical Considerations

The study was approved by the institutional Clinical Research Ethics Committee and conducted in accordance with the Declaration of Helsinki and relevant international guidelines. Data protection measures complied with the General Data Protection Regulation (EU 2016/679) and Spain’s Organic Law 3/2018 on Personal Data Protection. All participants were informed of their rights to access, rectify, or delete their data, as well as to limit or request the portability of their personal information.

## 3. Results

Over an eight-month data collection period, a total of 2262 haemodialysis sessions were analysed from 22 patients. Of these, 54.5% (n = 12) were male, with a mean age of 60.9 years (SE = 21.4). Within the cohort, 27.3% were diagnosed with diabetes mellitus and 81.8% with arterial hypertension (Table 1). The most commonly employed dialysis modality was post-dilution haemodiafiltration, which was used in 62.8% (n = 1419) of sessions.

Table 2 provides a detailed overview of the 2262 dialysis sessions, focusing on the incidence of intradialytic hypotension, biosensor usage, and a range of clinical and technical parameters.

A total of 279 sessions (12.3%) were associated with intradialytic hypotension, while the remaining 1983 sessions (87.7%) did not present hypotensive events. Biosensors were employed in 64.6% of sessions (n = 1464), whereas 35.3% (n = 798) were conducted without biosensor support. Among the sessions in which biosensors were used, 61.4% utilised the Blood Volume Monitor (BVM), while 38.6% employed the Haemomaster device.

With respect to the treatment modality, 37.2% of sessions were conducted using high-flux haemodialysis, while 62.8% employed post-dilution haemodiafiltration. The average pre-dialysis weight was 63.8 kg (SE = 15.4), and the mean ultrafiltration volume was 2.0 L (SE = 0.9). Post-dialysis weight averaged 62.0 kg. The mean heart rate decreased from 78.5 beats per minute (bpm) before dialysis to 73.5 bpm after dialysis. 

The comparison between sessions with and without biosensor monitoring (Table 3) revealed several statistically significant differences. Most notably, the incidence of intradialytic hypotension was lower in sessions monitored with biosensors (11.2%) compared to those without (*p* = 0.021). 

In the Nikkiso system, the **dVS (Dynamic Volume Status)** parameter represents the relative blood volume; that is, the percentage variation in blood volume during the haemodialysis session, monitored in real time to control ultrafiltration and prevent hypotension. It is expressed as a percentage change with respect to the patient’s initial blood volume.

In Fresenius, the **VCM (Volumetric Control Module)** functions as a blood volume monitor that also measures relative changes in blood volume, expressed as a percentage, to adjust ultrafiltration and maintain haemodynamic stability. Although the exact term may vary depending on the model, the unit is likewise a percentage.

Although replacement volume (22.5 L vs. 21.8 L) and pre-dialysis weight (65.6 kg vs. 66.2 kg) were similar between groups, interdialytic weight gain differed significantly (1.8 L vs. 2.0 L; *p* < 0.001), with lower gains observed in patients undergoing sessions with biosensor monitoring.

Regarding haemodynamic parameters, patients in the biosensor group exhibited significantly higher systolic blood pressure both before (136.9 mmHg vs. 129.9 mmHg) and after dialysis (137.4 mmHg vs. 129.0 mmHg), with both comparisons yielding *p*-values < 0.001. No significant differences were observed in diastolic blood pressure either pre-dialysis (*p* = 0.931) or post-dialysis (*p* = 0.122). However, mean arterial pressure was significantly higher in the biosensor group both before and after the session (*p* < 0.001 for both) (Table 2).

The target Kt remained comparable between groups (55.4 vs. 55.6; *p* = 0.841) (Table 2). However, a statistically significant difference was observed in patient age, with individuals in the biosensor group being slightly older (62 years vs. 59 years; *p* < 0.001).

Further subgroup analysis of sessions associated with hypotension revealed that these patients had lower mean values in the VCM parameter (87.4 vs. 84.1; *p* = 0.010). In contrast, differences in DVS were not statistically significant (*p* = 0.651). Additionally, the distribution of biosensor types—Blood Volume Monitor (BVM) versus Haemomaster—did not differ significantly between hypotensive and non-hypotensive sessions (*p* = 0.531) (Table 3).

Importantly, the use of biosensors was significantly associated with a lower incidence of hypotensive events (*p* = 0.021). Among sessions in which hypotension occurred, only 11.2% were monitored using biosensors, whereas 85.6% of sessions without hypotension involved biosensor monitoring (Table 3).

Furthermore, biosensor use was associated with several clinical and technical parameters, including treatment modality, blood pressure trends, and interdialytic weight gain. These findings highlight the potential of biosensor-guided monitoring to improve haemodynamic stability and optimise fluid management in patients undergoing chronic haemodialysis.

Finally, male sex and the presence of diabetes mellitus were negatively associated with biosensor use, while age did not show a significant association (Table 4). This multivariate analysis was conducted to identify potential confounding factors that may have influenced biosensor allocation, given the non-randomised nature of the study. Detecting such associations is essential to assess the presence of selection bias and to improve the interpretation of the clinical outcomes observed.

## 4. Discussion

This study analysed 2262 haemodialysis sessions from 22 patients with advanced chronic kidney disease, reporting an overall intradialytic hypotension (IDH) incidence of 12.3%. The implementation of biosensors in 64.6% of these sessions enabled a comprehensive comparison between biosensor-assisted treatments and standard clinical care. The results revealed significant clinical differences between groups, particularly regarding the frequency of hypotensive episodes, blood pressure trends, and interdialytic weight gain, reinforcing the role of biosensors in enhancing volume control and haemodynamic stability.

The observed hypotension rate aligns with findings from previous studies, which estimate an incidence ranging from 5% to 30%, depending on population characteristics, dialysis modality, and the diagnostic criteria applied for hypotension [15,16]. This variability is consistent with differences in clinical practice, prescription strategies, and patient comorbidity profiles. In our cohort, 81.8% of patients had hypertension and 27.3% had diabetes mellitus, both recognised risk factors for haemodynamic instability during haemodialysis [17].

The integration of biosensors—namely, the Blood Volume Monitor (BVM) and the Haemomaster—into dialysis sessions was associated with a statistically significant reduction in the incidence of hypotensive events. These findings support the hypothesis that continuous monitoring of relative blood volume enables real-time optimisation of ultrafiltration and dialysate conductivity, thereby preventing abrupt vascular volume depletion [18,19]. This is consistent with prior studies demonstrating that blood volume monitoring reduces both the frequency and severity of IDH, especially among high-risk populations [20,21,22]. Notably, a randomised crossover trial conducted by Leung et al. demonstrated that BVM-guided ultrafiltration significantly reduced the occurrence of IDH episodes, providing direct evidence of its clinical utility in standard haemodialysis settings [15].

Patients monitored with biosensors also demonstrated more favourable blood pressure profiles, with significantly higher systolic and mean arterial pressures both pre- and post-dialysis. These findings suggest improved cardiovascular stability during sessions. The ability of biosensors to detect early haemoconcentration facilitates proactive adjustments in fluid removal or dialysate composition, thereby preventing excessive intravascular depletion [23].

In addition, interdialytic weight gain was significantly lower among patients treated with biosensor-guided sessions. This may reflect more precise fluid management supported by real-time feedback, allowing for tighter control of fluid status. Reduced interdialytic weight gain is associated with lower cardiovascular stress and improved long-term outcomes in haemodialysis patients [24,25]. Importantly, this difference was achieved without compromising dialysis adequacy, as indicated by comparable target Kt values, supporting the safety and efficacy of biosensor-guided volume management.

The predominance of post-dilution haemodiafiltration (62.8% of sessions) is also notable. This modality has shown superiority over conventional high-flux haemodialysis in terms of solute clearance and cardiovascular protection, particularly when combined with biofeedback technologies [26,27]. Our findings suggest that biosensor implementation is not only feasible within advanced dialysis modalities, but may also amplify their clinical benefits by providing real-time haemodynamic control.

From a physiological perspective, the improvements observed in blood pressure regulation and fluid balance may be attributed to the biosensors’ role in supporting the vascular refilling capacity. During ultrafiltration, plasma water shifts from the interstitial to the intravascular compartment to preserve circulating volume. However, in many patients—particularly those with autonomic dysfunction, diastolic impairment, or endothelial dysfunction—this compensatory mechanism is compromised [28]. Biosensors detect impaired refill patterns through haemoconcentration signals, allowing for early clinical intervention before critical hypotension occurs [29].

Although the specific type of biosensor (BVM vs. Haemomaster) was not independently associated with IDH incidence, this may reflect the fact that both systems operate on similar optical or ultrasonic principles for detecting changes in blood volume. Nonetheless, performance may vary depending on patient-specific variables such as haematocrit, vascular access type, or intradialytic dynamics, which warrants further investigation in device-specific trials [16,30]. While the incidence of hypotension did not significantly differ between devices, sessions with hypotension exhibited significantly lower average values in the VCM of the Fresenius system. This may suggest a greater sensitivity to early volume depletion. In contrast, the Haemomaster system, which uses DVS, did not show differentiation between hypotensive and stable sessions. These preliminary findings may indicate differing sensitivity thresholds or response kinetics between devices. Further head-to-head studies are required to determine which biosensor offers faster detection, greater sensitivity, and optimal usability for nursing staff in real-world dialysis settings.

Interestingly, multivariate analysis revealed that male sex and the presence of diabetes mellitus were negatively associated with biosensor use. These findings raise important questions regarding potential disparities in access or decision making related to biosensor application. Although speculative, this may reflect perceived differences in tolerability, baseline haemodynamic risk, or comorbidity-based prioritisation. These results underscore the importance of ensuring equitable access to advanced monitoring technologies across all patient subgroups.

### 4.1. Strengths and Limitations

One of the main strengths of this study lies in the volume of dialysis sessions analysed, which provides robust observational data derived from real-world clinical practice. The study design allowed for comparison between two well-defined time periods, facilitating the assessment of biosensor implementation under routine care conditions. The use of consistent definitions of hypotension and the systematic collection of key haemodynamic and treatment-related variables further reinforces the validity of the findings.

While the quasi-experimental design does not permit definitive causal inferences, it reflects the complexity and pragmatic nature of real-world clinical settings, where randomisation is not always feasible. In this context, biosensor implementation was influenced by routine clinical decisions and device availability, which may have introduced confounding factors such as individual patient characteristics, session timing, or clinician discretion. Despite these limitations, multivariate analyses were conducted to explore relevant associations (Table 4), offering a solid preliminary evaluation of biosensor effectiveness in everyday practice. Although repeated-measures models and subgroup analyses were not applied—areas to be addressed in future studies—this design provides valuable insights into the performance and integration of biosensors in the routine management of haemodialysis.

### 4.2. Future Perspectives

These findings contribute to the growing body of evidence supporting biosensor use in haemodialysis. Future research should focus on multicentre, prospective randomised controlled trials with adequate power to detect differences in patient-centred outcomes, including cardiovascular events and mortality. It would also be valuable to examine cost-effectiveness, implementation barriers, and patient-reported experiences with biosensor-guided therapy. Incorporating artificial intelligence algorithms into biosensor feedback systems may further enhance their predictive capacity and enable personalised dialysis prescriptions based on dynamic risk modelling.

## 5. Conclusions

The implementation of volume-controlled biosensors in chronic haemodialysis has shown a clear association with a reduced incidence of intradialytic hypotension and improved haemodynamic stability. These technologies facilitate real-time monitoring and dynamic adjustment of treatment parameters, offering an effective strategy for individualised fluid management.

By enabling more precise control of ultrafiltration and vascular refill, biosensors contribute to the prevention of critical blood pressure drops and may improve overall cardiovascular tolerance to dialysis. The findings of this study support the integration of biosensor systems as a valuable clinical tool in the routine care of haemodialysis patients, particularly those at high risk of haemodynamic complications.

## Figures and Tables

**Table 1:** Description of the sociodemographic variables and comorbidities of the study population.

Patients Under Study, N = 22		
Age ($\bar{x}$, SE)		60.9 (21)
Gender (n; %)	Male	12 (54.5)
	Female	10 (45.5)
Patients with antihypertensive medication (n; %)		8 (36.4)
Diabetes Mellitus (n; %)		6 (27.3)
Arterial Hypertension (n; %)		18 (81.8)
Peripheral Vascular Insufficiency (n; %)		3 (13.6)
Cardiovascular Disease (n; %)		11 (50)
Cerebrovascular Accident (n; %)		0 (0)

**Table 2:** Influence of volume control biosensor use on demographic, haemodynamic, and haemodialysis treatment variables.

Use of Volume Control Biosensor				
		N = 2262		
		YES n = 1464	NO n = 798	*p*-Value
Hypotension episode (n; %)		164 (11.2)	115 (14.2)	0.021
Treatment modality (n; %)	Post-Dilution Haemodiafiltration	402 (27.5)	439 (55.1)	0.000
	High-Flux Haemodialysis	1061 (72.5)	358 (44.9)	
Replacement volume (litres) ($\bar{x}$, SE)		22.5 (5.1)	21.8 (4.6)	0.086
Pre-haemodialysis weight ($\bar{x}$, SE)		65.6 (15.6)	66.2 (14.7)	0.576
Interdialytic weight gain (litres) ($\bar{x}$, SE)		1.8 (0.9)	2 (1.1)	0.000
Total ultrafiltration (litres) ($\bar{x}$, SE)		2.0 (0.8)	2.1 (0.9)	0.001
Post-haemodialysis weight (kg) ($\bar{x}$, SE)		60.8 (15.4)	64.2 (14.6)	0.379
Pre-haemodialysis heart rate (bpm) ($\bar{x}$, SE)		77.5 (12.6)	80.4 (13.3)	0.535
Post-haemodialysis heart rate (bpm) ($\bar{x}$, SE)		71.6 (12.6)	77.0 (13.8)	0.026
Pre-haemodialysis systolic blood pressure (mmHg) ($\bar{x}$, SE)		136.9 (30)	129.9 (28.7)	0.000
Post-haemodialysis systolic blood pressure (mmHg) ($\bar{x}$, SE)		137.4 (31.7)	129 (33.8)	0.000
Pre-haemodialysis diastolic blood pressure (mmHg) ($\bar{x}$, SE)		68.6 (18.5)	68.6 (17.8)	0.931
Post-haemodialysis diastolic blood pressure (mmHg) ($\bar{x}$, SE)		69.3 (19.9)	68 (18.2)	0.122
Pre-haemodialysis mean arterial pressure (mmHg) ($\bar{x}$, SE)		91.4 (20.4)	88.6 (20.6)	0.000
Post-haemodialysis mean arterial pressure (mmHg) ($\bar{x}$, SE)		91.1 (23.7)	84.1 (28.8)	0.000
Blood flow rate (ml/min) ($\bar{x}$, SE)		387.4 (37.1)	387.1 (35.7)	0.402
Target Kt ($\bar{x}$, SE)		55.4 (12.3)	55.6 (12)	0.841
Age (years) ($\bar{x}$, SE)		62 (21)	59 (20)	0.004
Gender (n; %)	male	683 (46.7)	461 (57.8)	0.000
	female	781 (53.3)	337 (42.2)	
Diabetes mellitus (n; %)		442 (30.2)	279 (35)	0.020
Arterial hypertension (n; %)		1190 (81.3)	636 (79.7)	0.360
Peripheral vascular insufficiency (n; %)		256 (17.5)	108 (13.5)	0.015
Cardiovascular disease (n; %)		681 (46.5)	401 (50.3)	0.089

Beats per minute (bpm). Millimetres of mercury (mmHg).

**Table 3:** Association of volume control biosensor usage parameters on hypotension episodes during haemodialysis sessions.

Hypotension Episode				
		YES	NO	*p*-Value
Dynamic Volume Status (%) ($\bar{x}$; SE)		10.5 (4.5)	10.7 (4.3)	0.654
Volumetric Control Module (%) ($\bar{x}$; SE)		87.4 (5.8)	84.1 (5.1)	0.000
Type of biosensor (n; %)	Blood Volume Monitor	97 (10.8)	803 (89.2)	0.531
	Haemomaster	67 (11.8)	499 (88.2)	
Biosensor usage (n; %)	Yes	164 (11.2)	1300 (88.8)	0.021
	No	115 (14.4)	683 (85.6)	
Treatment modality (n; %)	Post-Dilution Haemodiafiltration	156 (6.9)	1253 (55.9)	0.011
	High-Flux Haemodialysis	123 (5.4)	718 (31.8)	

**Table 4:** Influence between the use of a biosensor and various predictor variables, including age, sex (male), and the presence of diabetes mellitus among study participants.

Biosensor Usage				
	OR	CI		*p*-Value
Age	0.75	0.58	0.97	0.321
Sex (male)	0.73	0.56	0.95	0.010
Diabetes mellitus	0.75	0.58	0.98	0.030

## Data Availability

The data presented in this study are available upon reasonable request from the corresponding author. Due to privacy and ethical restrictions, individual-level data cannot be shared publicly.
